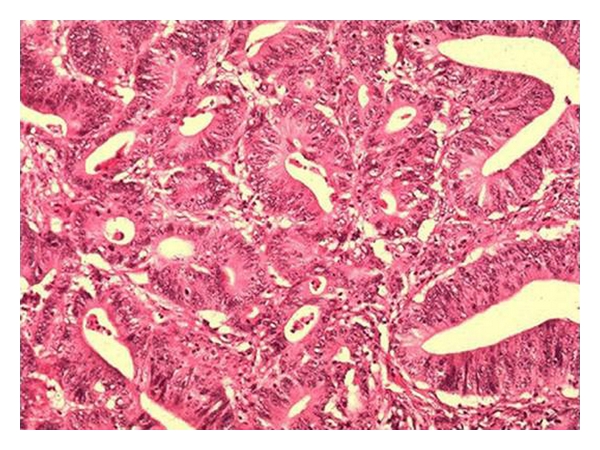# An Unusual Presentation of Metastatic Colon Adenocarcinoma in the Oral Cavity

**DOI:** 10.1155/2011/357518

**Published:** 2011-07-07

**Authors:** Abhilash Bhaskaran, Sam Harding, David Courtney

**Affiliations:** Maxillofacial Department, Derriford Hospital, Derriford, Plymouth, Devon PL6 8DH, UK

## Abstract

*Background*. A case report of a nonhealing ulcer of the tongue histologically proven to be adenocarcinoma. *Method*. A 92-year-old man underwent clinical, immunochemistry, and imaging investigation. *Results*. Tests confirmed a distant metastasis of a primary colorectal carcinoma. *Conclusion*. Metastasis from colorectal carcinoma to the oral cavity is primarily to bone, but non-healing ulcers of the oral cavity should be considered in differential diagnoses.

## 1. Introduction 

Oral carcinoma is the eighth most common form of cancer in the United Kingdom. A nonhealing ulcer of the oral cavity should always be treated with a high index of suspicion for malignancy. Although distant metastasis from primary neoplasms present elsewhere in the body to the oral cavity is rare [1], one should always consider this possibility. An intraoral ulcer could be the first clinical sign of a distant tumour. We report a case of oral metastasis resulting from a primary colorectal adenocarcinoma.

## 2. Case Report

**Figure 1:**
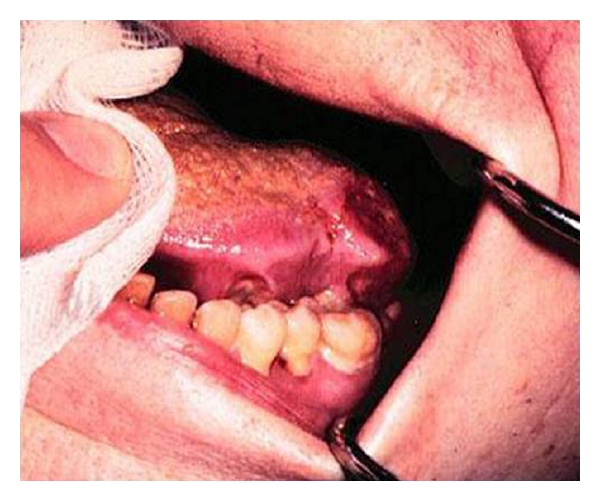


A 92-year-old man was referred to the department of oral and maxillofacial surgery on a two-week wait basis for assessment of an ulcer on the tongue (Figure 1). History revealed that the ulcer had been present for two months, was painful and causing discomfort while swallowing. The patient had lost seven kilograms of weight in one month, and he attributed this to the inability to eat due to pain. He had no significant relevant medical history. Social history revealed that he had been a pipe smoker for many years and took moderate amounts of alcohol on a regular basis. 

Clinical examination of the head and neck region revealed a 3 cm diameter indurated ulcer of the left posterior dorsum of the tongue. This ulcer was highly suspicious of malignancy. Examination of the neck revealed an ipsilateral solitary lymph node of 4 cm diameter in level 3 region. The patient was informed of the possible diagnosis of squamous cell carcinoma. He underwent urgent incisional biopsy under local anaesthesia, and the histopathology report was suggestive of an adenocarcinoma (Figure 2). Additional immunohistochemistry performed on the specimen demonstrated that the adenocarcinoma expressed cytokeratin 20 but lacked cytokeratin 7. In addition, the large bowel marker CDX2 was positive, whereas the pulmonary marker TTF1, the thyroid marker, thyroglobulin, and the prostate marker, PSA, were all negative. This suggested that the presenting oral lesion was the first sign of a possible distant metastasis of a primary bowel carcinoma. 

The patient underwent further investigations; chest X-ray, MRI head and neck, and CT chest and abdomen. The CT demonstrated a sigmoid carcinoma with local lymphadenopathy and also evidence of distant metastasis to right axillary, hepatic, intrapulmonary, retroperitoneal, brain, and bony disease to the thoracic vertebra. This patient was discussed in the head and neck multidisciplinary team meeting and subsequently referred to colorectal team for treatment. The colorectal team confirmed the presence of rectosigmoid tumour on colonoscopy that proved histologically to be a poorly differentiated adenocarcinoma. The patient was referred for palliation due to the extensive nature of his disease.

## 3. Discussion 

It is well documented in the literature that up to one percent of oral malignancies is a distant metastasis of primary tumours elsewhere in the body [1], but more commonly lungs in men and breast in women [2]. Most often these metastasises present as bony lesions. Colorectal cancers commonly metastasise to the liver or lungs [3], and it is not uncommon that multiple sites are involved before the oral presentation. This case was unusual in that the oral lesion was the first presenting sign of the tumour. 

An indurated and nonhealing ulcer of the oral cavity should always be looked upon with an eye of suspicion. Although the awareness of cancer has improved within the general population it is not uncommon for patients to present with oral ulcers that have been present for many months. The differential diagnoses of nonhealing and indurated ulcers commonly include; trauma, malignancies such as squamous cell carcinoma and lymphoma, primary syphilis and drug induced by drugs such as Nicorandil. Many of these ulcers are completely painless, and hence patients do not seek advice until the disease has progressed to an advanced stage. 

Metastasis from a colorectal carcinoma to the oral cavity is primarily to the bone [4], but there have also been a few case reports of oral metastasis to soft tissue sites such as the gingival [2]. In the presented case the substantial weight loss led to the suspicion of metastasis from a distant primary carcinoma. A review of the literature shows that oral metastatic tumours are uncommon and comprise approximately 1% of all malignant oral neoplasms [5, 6]. Most lesions are in the jawbones, and only 16% are in soft tissue, such as the gingival tissue [7]. With advances in immunohistochemistry it is now possible to accurately diagnose the site of primary tumour, aided by CT and MRI scanning. 

In summary, although rare, metastatic tumours should be included in the differential diagnosis of intraoral indurated soft tissue ulcers. Early detection of the type of malignancy and institution of appropriate treatment is of paramount importance in treatment outcome.

## Figures and Tables

**Figure 2:**